# The effect of probiotic supplementation on the clinical and para-clinical findings of multiple sclerosis: a randomized clinical trial

**DOI:** 10.1038/s41598-023-46047-6

**Published:** 2023-10-30

**Authors:** Kimia Motlagh Asghari, Neda Dolatkhah, Hormoz Ayromlou, Fatemeh Mirnasiri, Taher Dadfar, Maryam Hashemian

**Affiliations:** 1 Physical Medicine and Rehabilitation Research Center, Emam Reza Hospital, Tabriz University of Medical Sciences, Golgasht, Azadi Ave., Tabriz, Iran; 2 Faculty of Medicine, Tabriz University of Medical Sciences, Tabriz, Iran; 3 Department of Biology, School of Arts and Sciences, Utica University, Utica, USA

**Keywords:** Neurology, Nutrition

## Abstract

Multiple Sclerosis (MS) is a chronic demyelination disease of the central nervous system (CNS). The gut-brain axis involves communication between the nervous, endocrine, and immune systems. Probiotics can positively impact immune and inflammatory responses by regulating gut microbiota. A total of 40 MS patients (average age of 34.38 ± 6.65) were examined to determine the effect of the *Saccharomyces boulardii* supplement for four months compared to a placebo. The results showed that the *Saccharomyces boulardii* significantly decreased the inflammatory marker high-sensitivity C-reactive protein (hs-CRP) compared to the placebo (P < 0.001). The serum antioxidant capacity (TAC) also increased significantly in the probiotic group compared to the placebo (p = 0.004). Both the probiotic and placebo groups showed a reduction in the oxidative stress indicator malondialdehyde (MDA), but there was no significant difference between the two groups. Pain intensity (measured by Visual Analogue Scale) and fatigue severity (measured by Fatigue Severity Scale) significantly decreased in the probiotic group compared to the placebo (p = 0.004 and p = 0.01, respectively). The probiotic group experienced significant improvement in some quality of life scales (measured by 36-Item Short Form Survey) and somatic and social dysfunction subscale of General Health Questionnaire scores compared to the placebo group (p = 0.01). The study suggests that the *Saccharomyces boulardii* probiotic supplement may benefit inflammatory markers, oxidative stress indicators, pain, fatigue, and quality of life in MS patients.

## Introduction

Multiple Sclerosis (MS) is a chronic demyelination disease of the central nervous system (CNS)^1^. Women are three times more likely to develop MS than men, and it commonly affects those around age 30^2^. This condition disturbs individuals worldwide, with over 2.5 million people impacted globally^3^. MS can negatively impact patients' physical and mental health due to its chronic, unpredictable, and debilitating nature^4^. Currently, there is no conclusive cure for MS, and the present treatments can only manage its symptoms mainly by reducing the frequency of acknowledged attacks^5^. Therefore, the primary focus of MS care is managing the symptoms^6^.

Fatigue is a frequently experienced symptom of MS, which affects the patients' quality of life^7^. The treatment for fatigue is mainly based on trial and error, with limited success due to the various underlying mechanisms involved^7^. When dealing with MS, addressing the psychological challenges that patients often experience is essential^8^. One common symptom is central neuropathic pain, which can lead patients to seek treatment with opioids, Non-Steroidal Anti-Inflammatory Drugs (NSAIDs), antiepileptic drugs, and antidepressants^9^. However, these treatments may have minimal effectiveness with several side effects^10^. Recent research has shown a link between the gut microbiota and the central nervous system as the gut-brain axis, which involves communication between the nervous, endocrine, and immune systems^11^. Furthermore, studies have suggested that modifications to the gut microbiota can influence the inflammatory responses of individuals and animals with MS^12–14^.

Various clinical trials have demonstrated that probiotics can positively impact immune and inflammatory responses by regulating the gut microbial composition^15,16^. According to previous clinical trials, taking multi-strain probiotics containing at least two billion live microorganisms of *Bifidobacterium, Lactobacillus, Bacillus subtilis*, and *Streptococcus thermophiles* could lead to a significant reduction in serum inflammatory biomarkers such as C-reactive protein (CRP), Tumor necrosis factor alpha (TNF-α), and Interferon gamma (IFN-γ)^17^. A 12-week randomized controlled trial has revealed that taking probiotics can improve disability scores, mental health parameters (such as reduced depressive symptoms, anxiety, and stress), decrease inflammatory markers (such as hs-CRP), and oxidative stress (including plasma nitric oxide (NO) metabolites and malondialdehyde (MDA)), and enhance insulin resistance and cholesterol levels^18^. These findings indicate that probiotic supplementation could benefit various aspects of MS, including disability, mental health, inflammation, and metabolic conditions, by adjusting the gut microbiota.

Yeasts are a group of probiotics that provide various health benefits to the human body^19,20^. They could prevent and treat intestinal diseases, improve the immune system, and increase the absorption of minerals^21^. One yeast strain, *Saccharomyces boulardii (SB)*, in the category of *Saccharomyces cerevisiae species*, has been clinically proven to be an effective probiotic over five decades^19,20^. Certain strains of yeasts have received FDA approval for their potential use in improving human health^22^. Clinical trials and systematic reviews have extensively studied *SB*'s potential to cure various illnesses, including gastrointestinal disorders like irritable bowel syndrome, acute adult diarrhea, Crohn's disease, and giardiasis^23,24^. Nevertheless, we have not encountered any research confirming *SB*'s efficacy in treating patients with MS. The present study focuses on the impact of probiotic *SB* on inflammatory indexes and oxidative stress indicators in patients with MS. It is the first study to explore this topic. Additionally, the study examines how probiotic *SB* affects mental health, fatigue, pain, and quality of life in MS patients.

## Methods

### Trial design

This study was conducted as a 4-month prospective randomized double-blinded clinical trial among patients with a documented diagnosis of Relapsing–remitting MS (RRMS) referred to outpatient specialized and subspecialty clinics of Emam Reza Hospital of Tabriz University of Medical Sciences From June 2021 to March 2022 following the consolidated standards of reporting trials (CONSORT) guidelines. The eligible patients were randomly assigned to probiotic or placebo groups after a two-week wash-out period, with a 1:1 allocation ratio after baseline assessment. During the washing phase, patients were instructed to refrain from consuming probiotics such as yogurts with live active cultures, supplements, or any other dietary supplement, except for vitamin D3. The detailed trial protocol of the present study has been previously published^25^.

The trial was reviewed and approved by the ethics committee of the Research Vice-Chancellor of Tabriz University of Medical Sciences (IR.TBZMED.REC.1396.59) and was registered with the Iranian Clinical Trial Registry (IRCT20161022030424N1, 09/04/2018). Before any procedures were performed, we obtained written informed consent from all patients. Furthermore, we analyzed all data anonymously to ensure confidentiality.

### Participants

We used the convenience sampling method to recruit participants for our study. Female and male patients diagnosed with RRMS based on the revised McDonald criteria^26^ were referred to Emam Reza outpatient clinics and asked to participate if they met the eligibility criteria. The inclusion criteria included being between 18 and 55 years old, having an Expanded Disability Status Scale (EDSS) score of ≥ 4.5, not having any chronic diseases like hypertension or kidney/liver disorders, and not having experienced a relapse or changes in immunomodulatory therapy in the last three months as assessed by the enrolling investigator. Participants were excluded if they were pregnant or breastfeeding, smoked or consumed alcohol, had experienced acute gastrointestinal issues in the four weeks before enrollment, had any other musculoskeletal problems such as back pain, had received systemic glucocorticoid therapy in the last 30 days, or consumed other probiotics including yogurts with live, active supplements during the study.

### Interventions

The probiotic supplement, as well as the placebo, was provided by the TAKGEN ZIST, Tehran, Iran. *BioDigest®* is a commercial dietary supplement containing 250 mg of *SB (1010 CFU)*, a lactose filler, and a magnesium acetate oil. The control group received placebo capsules identical to *BioDigest* in shape, size, taste, smell, and other characteristics but lacked any microorganism content Patients were given either the probiotic "*BioDigest*" or a placebo capsule after lunch every day for over four months. We chose a 4-month intervention based on previous studies of the same probiotic supplement. Both groups were encouraged to take 1000 IU of vitamin D3 daily.

During the study, patients were instructed to take one capsule daily after a meal. They received a total of 14 every two weeks. Some patients were given fewer than 14 capsules in specific follow-up periods to monitor their adherence to the supplement. Patients who did not complete their intervention were excluded from the analysis at the end of each two weeks based on the number of capsules remaining. All patients continued their routine treatments for multiple sclerosis during the study. Acetaminophen tablets were permitted for pain relief, up to 2 g daily. However, we advised patients to avoid taking other anti-inflammatory analgesics or NSAIDs.

### Outcomes

#### Primary outcome

The primary outcome of the current study was the differences in mental health changes between the two groups due to the intervention.

#### Secondary outcome

The secondary outcomes were the mean difference in changes in fatigue, pain, quality of life, and biochemical parameters, including inflammatory and oxidative stress indices, between the two groups due to the intervention.

### Clinical measures

#### Mental health assessment

The General Health Questionnaire (GHQ-28) was utilized to assess mental health. Developed by Goldberg and Hiller in 1979, it consists of 28 questions divided into four subscales, each with seven questions. These subscales include physical symptoms, anxiety and sleep disorder symptoms, social function, and depression symptoms. The questionnaire has been extensively researched, and its Persian version is valid and reliable^27^.

#### Visual analogue scale (VAS)

We evaluated the amount of pain using the Visual Analogue Scale (VAS) at the start and end of our study. The VAS is a reliable pain scale that ranges from 0 to 10, with the intensity of pain assessed by asking the patients^28,29^.

#### Quality of life assessment

A widely-used tool called the 36-item short form (SF-36) questionnaire was used to measure the quality of life. This questionnaire is popular due to its comprehensive and concise nature, worldwide. It consists of 36 questions across eight dimensions, namely physical functioning, physical pain, general health, sense of activity, mental health, limitation of functioning due to emotional or physical issues, and social functioning. The scoring system ranges from 0 to 100 for each dimension, with higher scores indicating better health status^30^. Numerous studies have demonstrated the validity and reliability of this questionnaire. The Persian version of the questionnaire has also been confirmed to be valid^31^.

### Biochemical parameters assessment

#### High sensitivity CRP (hs-CRP)

Utilizing a particle-enhanced immunological turbidity test, the serum hs-CRP level was measured using the Pars Azmoon kit. The test was conducted with the BS-200 Chemistry Analyzer, manufactured in China by Shenzhen Mindray Bio-Medical Electronics Co Ltd.

#### Total antioxidant capacity (TAC)

The level of serum TAC was determined using the Naxifer TM kits made by Novin Navand Salamat Pishtaz Co. (located in Urmia, Iran). The ELISA reader from BioTek Instruments, Inc. (USA) was utilized following the manufacturer's guidelines.

#### Malondialdehyde (MDA)

Thiobarbituric acid (TBA) reactivity was analyzed to measure the level of the MDA using spectrophotometric measurements. The resulting fluorescent adducts had CVs below 5%^32^. MDA measurement was performed using Nalondi TM commercial kits from Novin Navand Salamat Pishtaz Co. (Urmia-Iran) and an ELISA reader from BioTek Instruments, Inc. (USA). The test using this kit has internal and external reliability or accuracy of 5% and 92.6%, respectively. Trial outcomes remained consistent throughout the study.

### Sample size

The primary outcome of this study is to evaluate how the mental health of MS patients is impacted by taking probiotic *SB* supplements when compared to a placebo. According to a similar study by Kouchaki et al.^18^ in which theGHQ-28 was used as an outcome measure, considering 6.5 as the minimal clinically significant difference and assuming types I and II error rates of 5% (α = 0.05) and 10% (β = 0.1; power = 90%), respectively, the sample size was calculated at 20 patients for each group. An additional 10% per group was considered to compensate for non-response and drop-outs, with a final sample of 25 patients per group and 50 for the whole trial.

### Randomization and blinding

For this study, a trained independent statistician used Random Allocation Software for a randomized assignment with an allocation ratio of 1:1. Block sizes varied from 2 to 4. We stratified the randomization based on participants' body mass index (BMI), age, and type of medication. The random number list was securely stored until the study's end. The allocation was kept in consecutively numbered opaque envelopes to conceal the allocation process and the type of treatment or intervention from the chief investigator, assessors, statistician, and patients. As patients enrolled in the study, they were allocated their trial numbers. Probiotic and placebo supplements were identical in shape, size, taste, odor, and appearance.

### Baseline characteristics

We collected patients' personal, medical, and demographic information through face-to-face interviews using a standard questionnaire developed by researchers. The data gathered included age, education level, occupation, adherence to special diets, history of diseases, and supplement consumption. To ensure compliance during the study and prevent sample leakage, we contacted subjects weekly by phone. We also monitored for infectious and gastrointestinal diseases, drug complications, and side effects.

### Anthropometric information

We used a Seca height meter with a range of 0–220 cm and measurement accuracy of 1 mm to accurately measure the participant's height and weight. The participants stood next to a wall without shoes and with relaxed shoulders. We also used a digital Seca scale with a capacity of 220 kg and an accuracy of 100 g. The participant's minimum clothing was taken into consideration. Their weight in kilograms was divided by the square of their height in meters to determine their body mass index. According to World Health Organization standards, individuals with a BMI between 25 and 29.9 kg/m2 were categorized as overweight, whereas those with a BMI of 30 or greater were classified as obese.

### Food intake

During the course of the study, the participants were instructed to keep a record of their dietary intake for a period of 3 days on three separate occasions—prior to the intervention, 2 months after the intervention began, and at the conclusion of the study. A software program called Nutritionist IV (developed by First Databank, San Bruno, CA) was used to monitor the participants' daily consumption of energy, macronutrients, and micronutrients. This software had been adapted specifically for Iranian foods and incorporated a "Home Scales Guide."

### Blood sample collection

During the study, blood samples were collected at the beginning and end of the intervention. A laboratory expert at Imam Reza Hospital of Tabriz University of Medical Sciences conducted the blood collection, ensuring that all necessary precautions were taken to prevent hemolysis. The 10-ml fasting blood samples were then centrifuged for 15 min at 1000*g* (3000 rpm) within 30 min. After centrifugation, the plasma samples were separated into pyrogen-free tubes using pyrogen-free pipette tips and stored at − 80 °C until analysis. The biochemistry department at Tabriz University of Medical Sciences analyzed the samples. Each biochemical parameter was measured using a specific device to minimize errors.

### Statistical methods

To ensure accurate results, we conducted tests to check the normality of data distribution using the Kolmogorov–Smirnov and Shapiro-Wilks tests. We used various methods, such as the independent sample t-test/Mann–Whitney U test and Fisher's exact test, to detect differences between groups at baseline. We used the paired sample t-test before and after the intervention to test within-group differences in primary and secondary outcomes. The mean of quantitative variables across study groups was compared using the Analysis of Variance (ANOVA). We employed the analysis of covariance (ANCOVA), which accounts for baseline values and potential confounding factors, to detect differences in studied variables between groups during the trial. All statistical analyses were carried out using two-tailed tests, and p-values lower than 0.05 were deemed statistically significant. The Statistical Package for Social Science v19.0 (SPSS Inc., Chicago, Illinois, USA) was used to perform all statistical analyses. It's worth noting that all analysis was done while investigators were blinded.

### Ethical consideration

We followed the Helsinki Declaration for our study. Before participating, each patient provided written informed consent. The institutional review board and Ethics Committee of Tabriz University of Medical Sciences approved the study protocol (No. IR.TBZMED.REC.1396.592), and it was registered on the Iranian clinical trial registration website (http://www.irct.ir: IRCT20161022030424N1, 09/04/2018) by the Declaration of Helsinki and Good Clinical Practice guidelines.

### Adverse events

All participants were asked to report any health issues throughout the trial period. Potential adverse effects were assessed twice a month through phone check-ins and self-reports.

## Results

### Pre-Randomization characteristics

**Figure 1:**
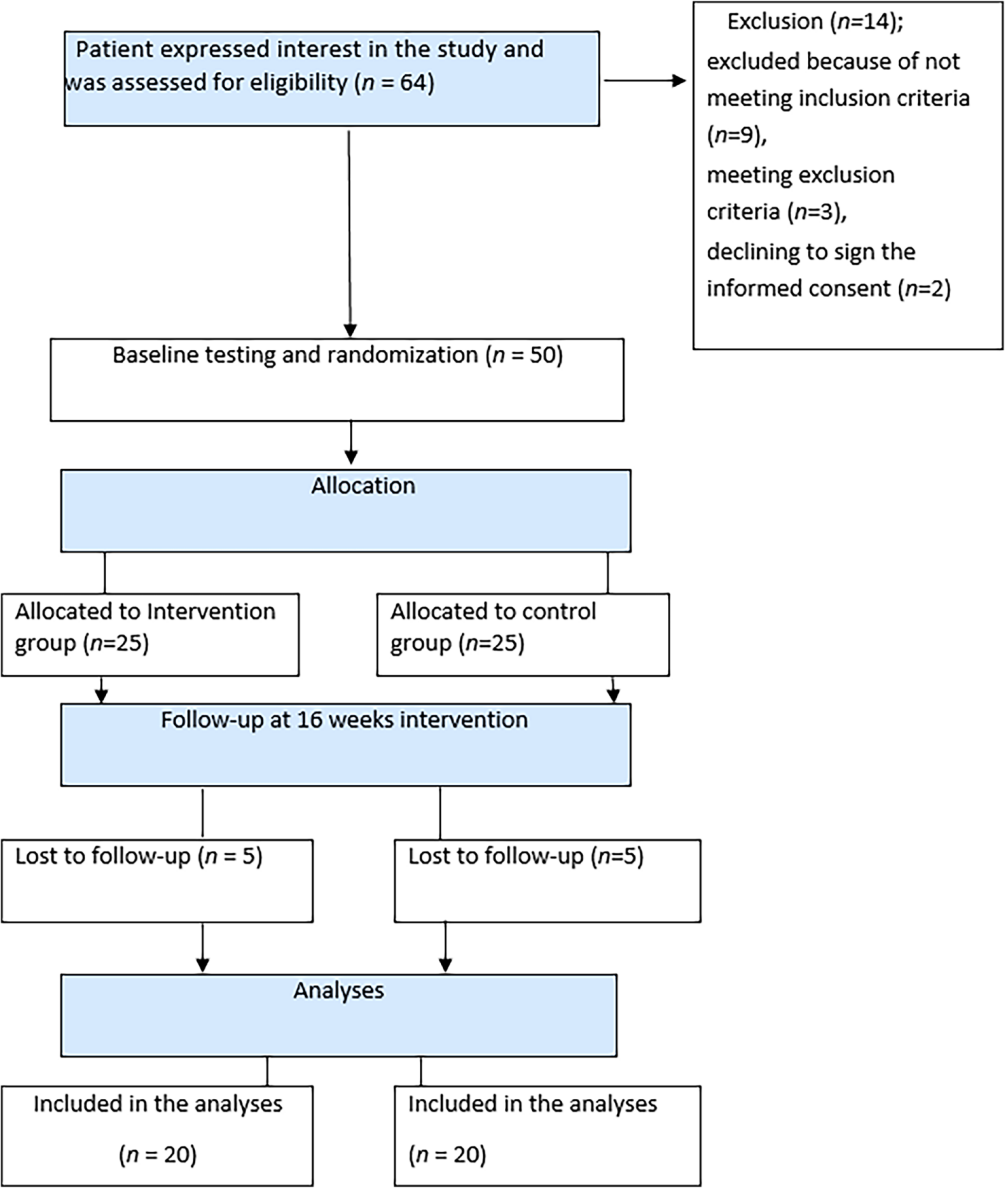
CONSORT flow of participants.

The study began recruiting participants on June 11, 2021; the last follow-up occurred on March 20, 2022. A flowchart in Fig. 1 illustrates that out of the 64 patients screened for participation, 14 were excluded. Nine patients did not meet the inclusion criteria, three met the exclusion criteria, and two declined to sign the informed consent. Ultimately, 50 patients were enrolled in the trial. Five patients from each group were lost to follow-up, and 40 patients completed the test and were included in the final analysis. The mean age in the probiotics and placebo groups was 33.80 ± 1.37 and 34.95 ± 7.03 years, respectively. Both groups had similar demographic and clinical parameters at baseline, as shown in Table 1.

**Table 1:** Baseline demographic and clinical characteristics of the participants in the two study groups at baseline.

Characteristics	Case (probiotic supplement group) N = 25	Control (placebo group) N = 25	p value*
Age (years), mean ± SD	33.80 ± 1.37	34.95 ± 7.03	0.591
Age (years), n (%)			
18–29	9 (36)	9 (36)	
30–39	13 (52)	13 (52)	
≥ 40	3 (12)	3 (12)	
Sex, n (%)			
Male	7 (28)	8 (32)	0.872
Female	18 (72)	17 (68)	
Education, n (%)			
Elementary	7 (28)	9 (36)	0.481
Diploma	12 (48)	9 (36)	
College	6 (24)	7 (28)	
Occupation, n (%)			
Unemployed	9 (36)	7 (28)	0.429
Employed	16 (64)	18 (72)	
Marriage status, n (%)			
Single/divorced/widowed	6 (24)	3 (12)	0.602
Married	19 (76)	22 (88)	
Living arrangements, n (%)			
Alone	3 (12)	1 (4)	0.901
With others	22 (88)	24 (96)	
Baseline expanded disability status scale score, Mean ± SD	3.15 ± 1.33	3.62 ± 0.88	0.105
Time since MS diagnosis, (years), mean ± SD	6.88 (4.55)	7.11 (5.91)	0.374
Previously corticosteroid use, n (%)			
No	9 (36)	8 (32)	0.682
3 months ago	3 (12)	1 (4)	
> 3 months ago	13 (52)	16 (64)	
Drug, n (%)			
Fingolimod	9 (36)	8 (32)	0.611
Natalizumab	11 (44)	10 (40)	
Glatiramer acetate	5 (20)	7 (28)	
Weight (kg), mean ± SD	72.25 ± 8.76	11.31 ± 2.52	0.745
Height (cm), mean ± SD	167.10 ± 6.86	165.35 ± 6.40	0.410
Body mass index (kg/m^2^), Mean ± SD	25.89 ± 2.96	25.99 ± 3.55	0.924
Body mass index (kg/m^2^)			
18.5–24.9	7 (28)	7 (28)	
25–29.9	12 (48)	12 (48)	
≥ 30	6 (24)	6 (24)	

*All values are presented as mean ± SD or n (%) and were analyzed using an independent sample t-test/ Mann–Whitney U test or Fisher's exact test.

After analyzing the dietary records gathered at the beginning, middle, and end of the intervention, it was discovered that there was no significant difference in the average dietary consumption between the two groups. Unfortunately, this data cannot be displayed at this time. We gathered data on the vitamin D3 intake of participants before the study and compared the two groups. Our findings indicate that there were no significant differences in the number of participants taking vitamin D3 before the study. We also monitored the participant's adherence to the recommended daily vitamin D3 supplementation of 1000 IU throughout the study. Our data showed that both groups were compliant with this recommendation, and there were no significant differences in terms of starting or discontinuing vitamin D3 supplementation between the groups.

### Primary outcome

The table shown as Table 2 summarizes the scores of the GHQ-28 subscales before and after the intervention for both placebo and probiotic groups. No significant differences were detected in the GHQ-28 subscale scores between the trial groups at the beginning (all p > 0.05). However, after treatment, there was a significant improvement in GHQ somatic (probiotic: 1.80 ± 1.90 vs. placebo: 0.10 ± 1.25, p = 0.01) and GHQ social dysfunction (probiotic: 1.60 ± 2.77 vs. placebo: 0.46 ± 2.29, p = 0.01) subscale scores in the probiotic group, which was significantly higher than the control group after adjusting to baseline variables (P < 0.05).

**Table 2:** Results of mental health (GHQ-28) in the studied patients (Case n = 20, Control n = 20).

Variable	Probiotic group N = 20	Placebo group N = 20	p**	p***
GHQ somatic, (points)				
Before intervention,	14.85 ± 4.19	14.55 ± 2.83	0.793	< 0.001
After intervention	16.65 ± 2.75	14.65 ± 2.36	0.021	
Mean changes*	**1.80 ± 1.90**	0.10 ± 1.25	0.004	
GHQ anxiety/insomnia, (points)				
Before intervention	16.15 ± 3.88	15.75 ± 3.22	0.725	0.719
After intervention	17.15 ± 2.96	16.70 ± 2.59	0.612	
Mean changes*	1.00 ± 2.99	0.95 ± 2.13	0.968	
GHQ social dysfunction, (points)				
Before intervention	14.20 ± 4.34	14.55 ± 4.04	0.743	0.041
After intervention	15.80 ± 2.54	15.01 ± 3.30	0.967	
Mean changes*	**1.60 ± 2.77**	0.46 ± 2.29	0.011	
GHQ depression, (points)				
Before intervention	18.75 ± 3.07	19.90 ± 1.48	0.497	0.508
After intervention	19.50 ± 1.79	19.55 ± 1.50	0.812	
Mean changes*	0.75 ± 2.42	− 0.35 ± 1.92	0.327	

All values are presented as mean ± SD.

*Paired-samples t-test (statistically significant values are shown in bold).

**One-way ANOVA.

***Analysis of covariance (ANCOVA) adjusted for baseline measures.

### Secondary outcomes

As shown in Table 3, there was no baseline difference concerning the pain intensity (probiotic: 3.75 ± 2.38 vs. placebo: 2.80 ± 2.37) and fatigue (probiotic: 28.50 ± 14.29 vs. placebo: 30.95 ± 13.20). After four months of probiotic intervention, the pain intensity based on VAS reduced significantly in the probiotic group compared to placebo after adjustment to baseline variables (probiotic: − 1.15 ± 0.67 vs. placebo: − 0.35 ± 0.81, p = 0.004). FSS decreased considerably in the probiotic group after four months of supplementation which was significantly higher than changes in the placebo group after adjustment to baseline variables (probiotic: − 5.55 ± 5.59 vs. placebo: − 2.35 ± 4.92, p = 0.01).

**Table 3:** Results of pain (VAS) and fatigue (FSS) in the studied patients (probiotic n = 20, placebo n = 20).

Variable	Probiotic group N = 20	Placebo group N = 20	p**	p***
VAS, (points)				
Before intervention	3.75 ± 2.38	2.80 ± 2.37	0.211	0.004
After intervention	2.60 ± 2.16	2.45 ± 2.03	0.862	
Mean changes*	**− 1.15 ± 0.67**	− 0.35 ± 0.81	0.005	
FSS, (points)				
Before intervention	28.50 ± 14.29	30.95 ± 13.20	0.577	0.010
After intervention	22.95 ± 10.86	28.60 ± 12.12	0.129	
Mean changes*	− **5.55 ± 5.59**	− 2.35 ± 4.92	0.020	

All values are presented as mean ± SD.

*Paired-samples t-test (statistically significant values are shown in bold).

**One-way ANOVA.

***Analysis of covariance (ANCOVA) adjusted for baseline measures.

Table 4 summarizes pre- and post-treatment scores on SF-36 scales and subscales for the probiotic and placebo groups. There were no baseline differences between trial groups concerning the quality of life scales and subscales. After 4 months, physical functioning scale (probiotic: 4.47 ± 8.22 vs. placebo: − 2.55 ± 7.18, p = 0.005), role limitation due to physical problems scale (probiotic: 3.75 ± 14.67 vs. placebo: − 5.00 ± 10.65, p = 0.016), social functioning scale (probiotic: 8.12 ± 12.35 vs. placebo: − 1.25 ± 5.59, p = 0. 043), vitality scale (probiotic: 4.50 ± 7.05 vs. placebo: 1.25 ± 11.50, p = 0.018), pain scale (probiotic: 9.75 ± 9.55 vs. placebo: 2.00 ± 9.71, p = 0.023), and physical health subscale (probiotic: 21.72 ± 26.15 vs. placebo: − 1.80 ± 27.27, p = 0.003) scores improved significantly in the probiotic group which was significantly higher than the placebo group after adjustment to baseline variables.

**Table 4:** Results of quality of life (SF-36) in the studied patients (Case n = 20, Control n = 20).

Variable	Probiotic group N = 20	Placebo group N = 20	p**	p***
SF-36—physical functioning scale, (points)				
Before intervention	81.77 ± 18.11	70.55 ± 27.29	0.192	0.005
After intervention	86.25 ± 16.61	68.00 ± 28.67	0.008	
Mean changes*	**4.47 ± 8.22**	− 2.55 ± 7.18	0.004	
SF-36—ROLE limitation due to physical problems scale, (points)				
Before intervention	87.50 ± 23.64	76.25 ± 35.79	0.565	0.016
After intervention	91.25 ± 20.31	71.25 ± 36.52	0.081	
Mean changes*	3.75 ± 14.67	− **5.00 ± 10.65**	0.108	
SF-36—role limitation due to emotional problems scale, (points)				
Before intervention	83.33 ± 31.53	73.22 ± 36.84	0.429	0.201
After intervention	88.24 ± 22.36	74.32 ± 35.62	0.301	
Mean changes*	**4.91 ± 19.57**	1.10 ± 24.18	0.799	
SF-36—social functioning scale, (points)				
Before intervention	71.25 ± 29.55	86.87 ± 16.95	0.121	0. 043
After intervention	79.37 ± 21.18	85.62 ± 16.85	0.414	
Mean changes*	**8.12 ± 12.35**	− 1.25 ± 5.59	0.015	
SF-36—vitality scale, (points)				
Before intervention	64.00 ± 26.43	65.25 ± 19.89	0.862	0.018
After intervention	68.50 ± 22.36	66.50 ± 15.96	0.653	
Mean changes*	**4.50 ± 7.05**	1.25 ± 11.50	0.022	
SF-36—emotional wellbeing scale (points)				
Before intervention	70.00 ± 25.01	75.00 ± 10.69	0.624	0.409
After intervention	73.80 ± 21.30	74.60 ± 15.58	0.870	
Mean changes*	**3.80 ± 8.65**	− 0.40 ± 14.84	0.192	
SF-36—general health perception scale, (points)				
Before intervention	68.75 ± 23.83	61.00 ± 19.37	0.242	0.516
After intervention	72.50 ± 17.50	64.75 ± 19.70	0.231	
Mean changes*	**3.75 ± 8.86**	3.75 ± 12.34	0.495	
SF-36—corporal pain scale, (points)				
Before intervention	70.37 ± 26.78	77.62 ± 19.87	0.620	0.023
After intervention	80.12 ± 19.90	79.62 ± 19.06	0.841	
Mean changes*	**9.75 ± 9.55**	2.00 ± 9.71	0.072	
SF-36—physical health subscale, (points)				
Before intervention	308.40 ± 76.59	285.42 ± 83.70	0.341	0.003
After intervention	330.12 ± 62.78	283.62 ± 86.88	0.063	
Mean changes*	**21.72 ± 26.15**	− 1.80 ± 27.27	0.008	
SF-36—mental health subscale, (points)				
Before intervention	288.58 ± 93.04	300.46 ± 60.52	0.762	0.105
After intervention	309.01 ± 71.74	301.06 ± 70.95	0.349	
Mean changes*	**20.42 ± 27.88**	0.60 ± 40.25	0.038	

All values are presented as mean ± SD.

*Paired-samples t-test (statistically significant values are shown in bold).

**One-way ANOVA.

***Analysis of covariance (ANCOVA) adjusted for baseline measures.

The serum samples were collected for additional measurements before and after the intervention. Table 5 summarizes the hs-CRP, TAC, and MDA data analysis results. After the 4-month probiotic intervention, the serum hs-CRP concentration decreased significantly in the probiotic group (p < 0.001). The decrease in the probiotic group after adjustment to baseline variables was higher than the placebo group (probiotic: − 2.60 ± 2.26 versus placebo: − 0.38 ± 1.42 μg/ml and p < 0.001). The level of MDA decreased significantly after four months of probiotic intervention (p = 0.049). These changes in the probiotic group were not different from the placebo group after adjustment to the baseline values (probiotic: − 0.54 ± 1.15 vs. placebo: − 0.43 ± 1.12 nmol/ml and p = 0.613).

**Table 5:** Results of inflammatory indexes and oxidative stress in the studied subjects (case n = 20, control n = 20).

Variable	Probiotic group N = 20	Placebo group N = 20	p**	p***
hs-CRP (g/mlµ)				
Before intervention	9.32 ± 2.04	8.62 ± 2.12	0.157	< 0.001
After intervention	6.72 ± 1.25	8.24 ± 1.67	0.174	
Mean changes*	**2.26 ± 2.60**	− 0.38 ± 1.42	0.001	
TAC (mmol/L)				
Before intervention	3.87 ± 1.28	3.12 ± 1.33	0.078	0.004
After intervention	4.38 ± 0.23	3.05 ± 1.02	0.001	
Mean changes*	0.51 ± 1.33	− 0.06 ± 1.22	0.163	
MDA (nmol/ml)				
Before intervention	5.36 ± 1.73	5.88 ± 1.61	0.332	0.613
After intervention	4.82 ± 1.78	5.44 ± 1.82	0.279	
Mean changes*	− **0.54 ± 1.15**	− 0.43 ± 1.12	0.774	

All values are presented as mean ± SD.

*Paired-samples t-test (statistically significant values are shown in bold).

**One-way ANOVA.

***Analysis of covariance (ANCOVA) adjusted for baseline measures.

The probiotic group showed an increase in TAC level while the placebo group demonstrated a decrease; nonetheless, these variances did not attain statistical significance (p = 0.103 and p = 0.817, respectively). After adjusting to baseline values, the changes observed in the probiotic group were significantly higher than in the placebo group (probiotic: 0.51 ± 1.33 versus placebo: − 0.06 ± 1.22 nmol/liter and p = 0.004).

### Compliance

Probiotics were well tolerated, and the patients reported no serious adverse effects or were recognized by the physicians. Compliance was high (greater than 95%). Of the 40 patients who completed the 4-month trial, the most common adverse effects included constipation (12.5%), weight gain (12.5%), nausea (10.0%), and worsened fatigue (5%). No patients showed evidence of MS relapse.

## Discussion

Studies examining probiotic intake and inflammatory indicators in MS are scarce, and their results are contradictory. The present study was the first to investigate the effects of *SB*, a probiotic, on inflammatory markers and oxidative stress indicators in patients with MS. The study also looked into the probiotic's impact on the mental health, fatigue, quality of life, and pain of MS patients. Forty MS patients were examined for four months to determine the effect of the *SB* supplement compared to a placebo. The probiotic group showed a significant decrease in the inflammatory marker hs-CRP level compared to the placebo. The serum TAC also increased significantly in the probiotic group compared to the placebo. Pain intensity and fatigue severity significantly decreased in the probiotic group compared to the placebo. The probiotic group also showed significant improvements in the quality of life scales and GHQ somatic and social dysfunction subscales compared to the placebo group.

Approximately 2.5 million people worldwide are affected by MS. MS is a chronic central nervous system disorder caused by an autoimmune reaction^3^. The condition is characterized by inflammation, demyelination, and neurodegeneration, which can lead to neurological symptoms and disabilities^33^. Recent studies have suggested that gut microbiota dysbiosis may contribute to the pathogenesis of MS and that probiotics—live microorganisms that confer health benefits to the host—may have a role in modulating the immune response and reducing inflammation in MS^34,35^.

According to research conducted by Kouchaki et al., probiotic supplementation with *Lactobacillus acidophilus, Lactobacillus casei, Bifidobacterium bifidum*, and *Lactobacillus fermentum* for 12 weeks improved serum levels of hs-CRP in patients with MS compared to a placebo^18^. Additionally, MDA serum levels also showed improvement in the subjects who received the probiotic supplements compared to those who received the placebo^18^. Tankou et al.^15^ conducted a study on 22 MS patients to observe the effects of probiotic supplementation containing *Lactobacillus, bifidobacterium*, and *Streptococcus*. Their study revealed that these probiotic supplementations for two months created an anti-inflammatory environment and reduced the frequency of intermediate monocytes (CD14highCD16low) in the immune response^15^.

In a clinical trial conducted by Rahimlou et al.^17^, the effectiveness of multi-strain probiotic supplementation *(Lactobacillus acidophilus, Lactobacillus casei, Lactobacillus rhamnosus, Lactobacillus bulgaricus, Bifidobacterium breve, Bifidobacterium longum*, and *Streptococcus thermophilus*) was investigated over six months. The study involved 70 patients with MS, and the results showed that probiotic supplementation led to a significant increase in brain-derived neurotrophic factor (BDNF) levels and a substantial decrease in the inflammatory factor IL-6 levels^17^. The researchers also concluded that the six-month probiotic supplementation improved mental health parameters^17^. The findings revealed that compared to the placebo, probiotic supplementation caused a significant improvement in the GHQ-28, Beck Depression Inventory-II (BDI-II), FSS, and Pain Rating Index (PRI)^17^. The present study aligns with Zamani et al*.*'s findings that taking probiotic capsules with *Lactobacillus acidophilus, Lactobacillus casei*, and *Bifidobacterium bifidum* for eight weeks can reduce hs-CRP serum levels in rheumatoid arthritis patients compared to a placebo^47^. C.H. Choi et al*.*^48^ conducted a study to assess the impact of *Saccharomyces boulardii* on quality of life and symptoms in patients suffering from diarrhea-predominant IBS or mixed-type IBS^48^. They discovered that *SB* enhanced the patients’ quality of life^48^. Shavakhi et al*.*^49^ also studied the effectiveness of a multi-strain probiotic species containing seven species of bacteria, including *Lactobacillus* and *Bifidobacterium*, in treating Iranian IBS patients. They found no significant advantages for this probiotic species over placebo in relieving IBS symptoms^49^. However, the probiotic group noted a statistically significant improvement in the quality of life score^49^.

Oxidative stress, a condition where reactive oxygen species (ROS) production exceeds the body's detoxification capability, is thought to be responsible for the development of MS^36^. Cellular components, such as lipids, proteins, and DNA, may be impaired by ROS, which can result in inflammation, demyelination, and neurodegeneration^36^. Malondialdehyde (MDA) is a byproduct of lipid peroxidation and is a reliable marker of oxidative stress-mediated lipid damage^37^. Studies have confirmed a dysregulated MDA level, with the increased MDA level in cerebrospinal fluid (CSF) in MS patients compared to the healthy controls. These conditions positively correlated with disease severity^37,38^. These findings suggest that MDA could be a noteworthy biomarker for oxidative stress in MS pathogenesis and disease disability.

**Figure 2:**
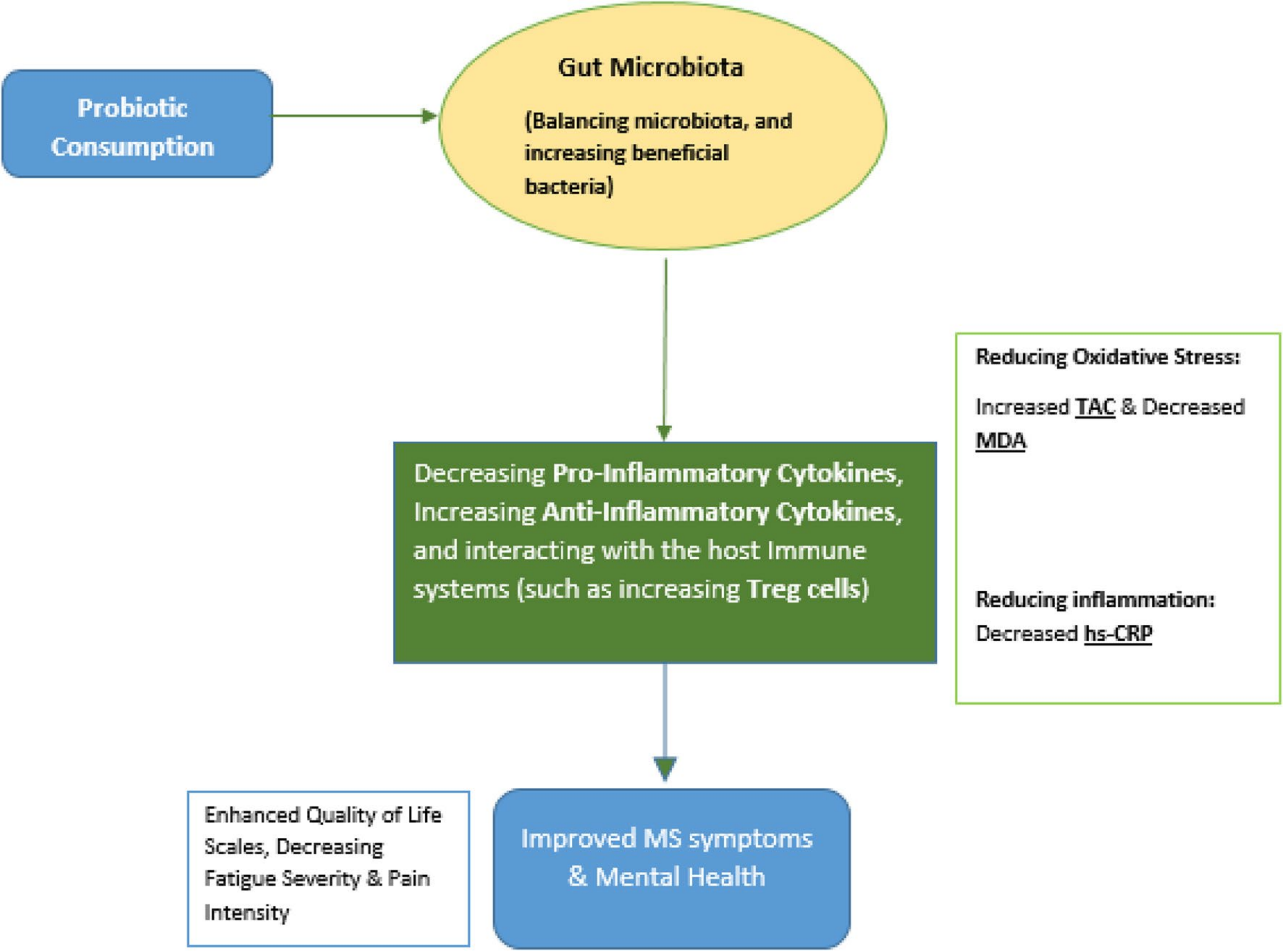
Proposed mechanism of probiotic impact on health of MS patients.

In recent years, the gut-brain axis, a dynamic and bidirectional communication system between the gastrointestinal tract and the CNS, has garnered significant attention for its potential relevance to various neurological conditions, including MS^39^. Emerging evidence suggests that gut microbiota composition and function alterations can influence not only local gastrointestinal health but also immune responses and inflammatory processes throughout the body, potentially including those within the CNS^40^. The gut microbiota can impact the development of MS by affecting oxidative stress and inflammation^41–44^. Commensal bacteria in the gut possess anti-oxidative properties and can suppress inflammation, while pathogenic microbiota can induce inflammation and shift the redox balance towards oxidative stress^41^. Changes in the gut microbiota have been linked to increased oxidative stress, chronic neuroinflammation, and neurodegeneration^42^. Elevated levels of oxidative stress and inflammatory cytokines have been linked to a reduction in specific gut bacteria^43^. The microbiota present in the gut is regarded as an organic entity comprising microbes. Any alterations to this entity may result in the immune system becoming inflamed and activated^43^. A healthy gut microbiota has been found to have a significant antioxidative and anti-inflammatory effect^44^. The role of the gut microbiome in MS has gained interest due to the discovery that oxidative stress is a crucial element in the pathogenesis of the disease. While the exact mechanisms are not yet fully understood, research suggests that the gut microbiota could be a potential target for treating MS and other disorders^41–44^. Probiotics can be the missing component of diet interventions focusing on how the food matrix and diet contents interact with gut microbiota^45^. Therefore, specific probiotics and dietary interventions probably control the function of the intestinal barrier and local and systemic inflammation and reverse the defective cycle of inappropriate metabolic regulation^46^. While our study did not directly investigate the mechanistic link between the gut microbiota and MS symptoms, the potential impact of dietary intervention and probiotic supplementation on the gut microbiota is noteworthy. The dietary changes implemented in our study, although primarily focused on a restricted diet, may have indirectly influenced the gut microbiota composition. Human dietary patterns can shape the microbial community within the gut, and our related recommendations may have impacted the gut-brain axis by modulating the gut microbiota. While more research is needed to elucidate the mechanisms by which the gut-brain axis may be involved in MS pathogenesis and symptomatology, our study provides valuable insights and prompts further exploration into the interplay between dietary interventions, gut microbiota, and MS outcomes. The present study suggests that the *SB* probiotic supplementation may benefit inflammatory biomarkers, oxidative stress indicators, pain, fatigue, and quality of life in MS patients. We propose a mechanism (see Fig. 2) that illustrates the potential impact of probiotics on MS health. This diagram visually demonstrates how probiotic consumption may indirectly affect oxidative stress, inflammation, and other MS-related factors via modulation of the gut microbiota. The proposed mechanism suggests that probiotic consumption can create a chain of events that begins with alterations in gut microbiota, leading to decreased inflammation and oxidative stress. These changes, in turn, influence interleukins, cytokines, and the overall well-being of MS patients. The proposed mechanism provides a comprehensive explanation for the observed reductions in hs-CRP, MDA, pain, fatigue, and depression, as reported in our study findings. It highlights the potential of probiotics as a non-pharmacological approach to improve the quality of life for individuals with MS by targeting multiple factors contributing to the disease's progression and symptoms.

## Limitations and recommendations

The current clinical trial has shown that supplementing *SB*probiotic yeast for four months had beneficial effects on inflammatory indices and oxidative stress in MS patients. This intervention is simple, safe, effective, inexpensive, and accessible for adults with the disease. However, the study was conducted over a relatively short period, and further research is needed to fully understand the probiotic's mechanisms of action and determine its long-term effects. Extending the duration of the study might provide insights into the longer-term effects of probiotic supplementation and dietary changes. To improve future studies, it is suggested to conduct more extensive studies with higher sample sizes and examine other oxidative stress and lipid peroxidation indicators such as 2F-isoprostanes and oxidized LDL, blood lipids, and adipokines. Additionally, designing interventional studies, preferably including stool analysis to determine changes in stool composition (such as microorganisms and short-chain fatty acids), measuring circulating lipopolysaccharide, and evaluating the impact of simultaneous administration of probiotic and prebiotic species with different doses on nutritional status and indicators related to MS would be beneficial. On the other hand,by conducting subgroup analyses based on disease severity, age, or gender among MS patients, it would be possible to uncover differences in treatment response across different groups, thereby highlighting the variability in intervention outcomes.

It must be considered that conducting a 4-month prospective randomized double-blinded clinical trial with a restricted diet in an outpatient setting introduces additional limitations. Participants' adherence to the prescribed diet and medication regimen may have varied, and their exposure to external factors that can influence the results, such as environmental stressors and dietary triggers for MS symptoms, was not fully controlled. The study relied on self-reports from participants for dietary intake, medication adherence, and symptom assessment. This introduces the potential for recall bias, as participants may not accurately remember or report their activities and symptoms. Social desirability bias could also affect self-reported data. Moreover, maintaining the blinding of participants in an outpatient setting can be challenging. The placebo effect may have influenced participants' expectations and experiences, potentially affecting self-reported outcomes. The extent of this effect could not be precisely determined. Even though conducting dietary interventions in an outpatient setting is more practical and reflective of real-world scenarios for individuals with RRMS, the absence of an inpatient control group limits the ability to isolate and control variables that could influence the outcomes. MS is a complex condition affected by various factors, and the study did not have the advantage of inpatient control to minimize these confounding factors. The study prioritized real-world applicability in an outpatient setting, which led to certain limitations in control. This trade-off between generalizability and control should be acknowledged when interpreting the results.

## Conclusion

In conclusion, SB supplementation for four months decreased the serum level of hs-CRP and improved the serum levels of TAC compared to the placebo in patients with MS. Additionally, SB supplementation reduced the pain intensity and fatigue severity and improved some SF-36 quality of life scales and GHQ somatic and social dysfunction subscales compared to the placebo in MS patients.

The findings of this study suggest that *SB* may be a valuable adjunct therapy for controlling clinical symptoms, inflammation, and oxidative stress in MS patients. The probiotic's potential to improve mental health, fatigue, quality of life, and pain also highlights its valuable role in managing MS neuropsychological Symptoms. Further studies are to understand the probiotics' thorough mechanisms of action and their long-term effects.

## Data Availability

The data that support the findings of the present study are available upon reasonable request from the corresponding author. All relevant data generated and analyzed during the study, including clinical measurements, para-clinical assessments, and statistical analyses, will be made available to qualified researchers who wish to replicate or verify the results presented in the published manuscript.
